# circSMARCA5 Is an Upstream Regulator of the Expression of miR-126-3p, miR-515-5p, and Their mRNA Targets, *Insulin-like Growth Factor Binding Protein 2* (*IGFBP2*) and *NRAS Proto-Oncogene, GTPase* (*NRAS*) in Glioblastoma

**DOI:** 10.3390/ijms232213676

**Published:** 2022-11-08

**Authors:** Aurora Eliana Merulla, Michele Stella, Cristina Barbagallo, Rosalia Battaglia, Angela Caponnetto, Giuseppe Broggi, Roberto Altieri, Francesco Certo, Rosario Caltabiano, Marco Ragusa, Giuseppe Maria Vincenzo Barbagallo, Cinzia Di Pietro, Michele Purrello, Davide Barbagallo

**Affiliations:** 1 Department of Biomedical and Biotechnological Sciences, Section of Biology and Genetics “Giovanni Sichel”, University of Catania, Via Santa Sofia 89, 95123 Catania, Italy; 2 Department of Medical, Surgical Sciences and Advanced Technologies “G.F. Ingrassia”, Section of Anatomic Pathology, University of Catania, Via Santa Sofia 87, 95123 Catania, Italy; 3 Interdisciplinary Research Centre on the Diagnosis and Therapy of Brain Tumors, University of Catania, Via Santa Sofia 78, 95123 Catania, Italy; 4 Department of Medical, Surgical Sciences and Advanced Technologies “G.F. Ingrassia”, Neurological Surgery, Policlinico Rodolico-San Marco University Hospital, University of Catania, Via Santa Sofia 78, 95123 Catania, Italy

**Keywords:** circular RNA, circSMARCA5, microRNAome, microRNA processing, microRNA target, tumor suppressor, RNA binding protein, epigenetics

## Abstract

The involvement of non-coding RNAs (ncRNAs) in glioblastoma multiforme (GBM) pathogenesis and progression has been ascertained but their cross-talk within GBM cells remains elusive. We previously demonstrated the role of circSMARCA5 as a tumor suppressor (TS) in GBM. In this paper, we explore the involvement of circSMARCA5 in the control of microRNA (miRNA) expression in GBM. By using TaqMan^®^ low-density arrays, the expression of 748 miRNAs was assayed in U87MG overexpressing circSMARCA5. Differentially expressed (DE) miRNAs were validated through single TaqMan^®^ assays in: (i) U87MG overexpressing circSMARCA5; (ii) four additional GBM cell lines (A172; CAS-1; SNB-19; U251MG); (iii) thirty-eight GBM biopsies; (iv) twenty biopsies of unaffected brain parenchyma (UC). Validated targets of DE miRNAs were selected from the databases TarBase and miRTarbase, and the literature; their expression was inferred from the GBM TCGA dataset. Expression was assayed in U87MG overexpressing circSMARCA5, GBM cell lines, and biopsies through real-time PCR. TS miRNAs 126-3p and 515-5p were upregulated following circSMARCA5 overexpression in U87MG and their expression was positively correlated with that of circSMARCA5 (*r*-values = 0.49 and 0.50, *p*-values = 9 × 10^−5^ and 7 × 10^−5^, respectively) in GBM biopsies. Among targets, *IGFBP2* (target of miR-126-3p) and *NRAS* (target of miR-515-5p) mRNAs were positively correlated (*r*-value = 0.46, *p*-value = 0.00027), while their expression was negatively correlated with that of circSMARCA5 (*r*-values = −0.58 and −0.30, *p*-values = 0 and 0.019, respectively), miR-126-3p (*r*-value = −0.36, *p*-value = 0.0066), and miR-515-5p (*r*-value = −0.34, *p*-value = 0.010), respectively. Our data identified a new GBM subnetwork controlled by circSMARCA5, which regulates downstream miRNAs 126-3p and 515-5p, and their mRNA targets *IGFBP2* and *NRAS*.

## 1. Introduction

Glioblastoma multiforme (GBM) is the most common and aggressive malignant tumor among those affecting the central nervous system (CNS), with an average annual age-adjusted incidence rate of 3.23 per 100,000 individuals per year and a median overall survival of 14 months following diagnosis, according to the 2021 Central Brain Tumor Registry of the United States (CBTRUS) statistics [1]. The classification and diagnostic criteria of tumors of the CNS, published by the World Health Organization (WHO) in 2021, state that molecular signatures such as isocitrate dehydrogenase (IDH) mutational status, epidermal growth factor receptor (EGFR) gene amplification, telomerase reverse transcriptase (TERT) promoter mutation, and duplications and deletions occurring in chromosomes 7 and 10 need to be associated with specific histological and immunohistochemical features for a correct diagnosis of GBM [2]. Genomic features of GBM have been largely studied thanks to the data collected within The Cancer Genome Atlas (TCGA) [3]; accordingly, GBM can be classified into four molecular subtypes (classical, mesenchymal, proneural, and neural), based on specific gene expression profiles [4]. Non-coding RNA, especially microRNA (miRNA) expression profiles have also been used to classify GBM into different subtypes [5]. Intra- and inter-GBM tumor heterogeneity at different levels (cellular, molecular, and microenvironmental) defeat the effectiveness of therapeutical approaches currently used, leading to high rates of chemoresistance and relapse [6]. Therefore, GBM is still an incurable malignant tumor with less than 5% of individuals surviving 5 years after diagnosis.

Circular RNAs (circRNAs) are a recently discovered class of RNAs, mostly non-coding, characterized by covalently bound 5′ and 3′ termini [7,8]. The main biogenetic mechanism of circRNAs is back-splicing, occurring during pre-mRNA maturation [9]. CircRNAs are synthesized by almost all living organisms (apart from eubacteria) and by many viruses [10,11], following tissue- and developmental-specific patterns of expression, and appear particularly abundant in the brain [12]. The peculiar structure of circRNAs allows them to be more resistant to exonuclease-mediated digestion than their linear counterparts [13]. CircRNAs have been detected also in biological fluids, inside extracellular vesicles (EVs) [14,15,16], and, for these reasons, they may be used as stable non-invasive diagnostic, prognostic, or response-to-therapy biomarkers for several diseases, including GBM [17,18]. CircRNAs perform their function mainly by: (i) sponging miRNAs, being part of the competitive endogenous RNA (ceRNA) networks; (ii) interacting with RNA binding proteins (RBPs), defining their subcellular localization and modulating their activity; (iii) working as scaffold for transcription factors during the assembly of the pre-initiation complex in the first phase of RNA transcription [19]. Altered expression of different intra- and extra-cellular oncogenic or tumor-suppressive circRNAs has been linked to the pathogenesis and progression of several types of cancer [20].

CircSMARCA5 has been recently characterized as a downregulated tumor suppressor (TS) in GBM and additional tumors by ourselves and other researchers [21,22,23,24,25,26,27,28]; it also has been detected in serum-derived EVs, together with circHIPK3, and has been described as a good diagnostic candidate biomarker of GBM [17]. We also found that circSMARCA5 elicits its function at least in part by acting as a decoy for the pleiotropic serine- and arginine-rich splicing factor 1 (SRSF1) protein, therefore regulating its splicing activity [23].

MiRNAs belong to a large group of short (about 20-nucleotides long) non-coding RNAs, which act as post-transcriptional negative regulators of gene expression and whose sequences have been conserved during evolution [29]. The canonical pathway of miRNA biogenesis consists of the synthesis of large precursor molecules (pri-miRNA), followed by processing mediated by Drosha and Dicer enzymes that leads to the production of pre-miRNAs and mature miRNAs, respectively [30]. Similar to circRNAs, miRNAs are expressed according to tissue- and developmental-specific patterns and are involved in the regulation of many biological functions, including physiological and pathological conditions such as cancer [31,32,33,34,35,36,37]. The altered expression and function of miRNAs has been ascertained to play a critical role in the pathogenesis and progression of GBM [38,39,40,41,42,43,44].

To expand our knowledge of the pathways regulated by circSMARCA5, in this study we investigated its potential role as an upstream regulator of the microRNAome (miRNAome) in GBM cells.

## 2. Results

### 2.1. The miRNAome Expression Profile Is Dysregulated upon circSMARCA5 Overexpression in U87MG

**Figure 1:**
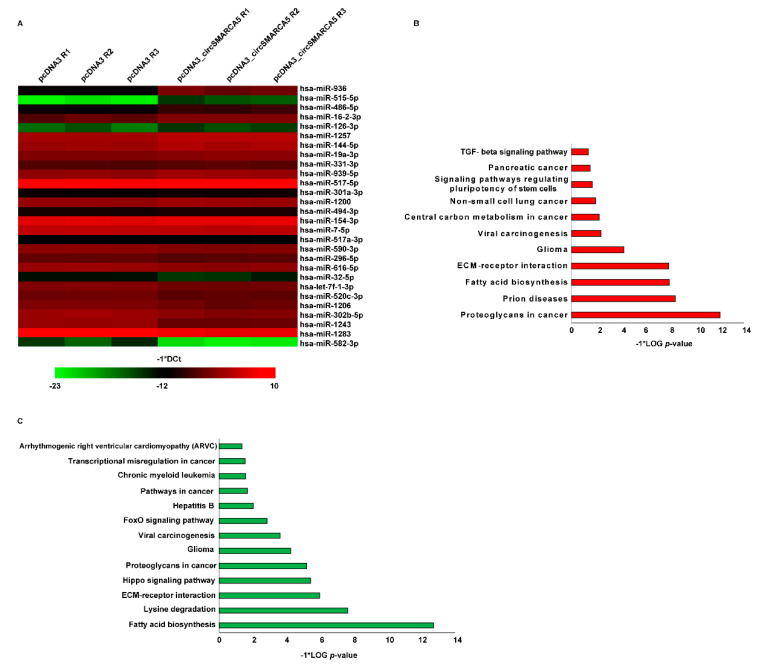
(**A**) Heatmap representation of the expression level of significant DE miRNAs in U87MG transfected for 24 h with the empty plasmid vector (pcDNA3) or with the plasmid vector expressing circSMARCA5 (pcDNA3_circSMARCA5). Data are represented as −1*DCt (the less its value, the less the expression of DE miRNAs; the higher its value, the higher the expression of DE miRNAs) for the three replicates (R1, R2, R3) of each experimental condition. (**B**,**C**) Enriched pathways (EPs) controlled by upregulated (**B**) and downregulated (**C**) miRNAs. Only significant (−1*LOG *p*-value ≥ 1.3) EPs are reported in the graphs.

To check for circSMARCA5-mediated regulation of the microRNAome in GBM cells, the expression of 748 miRNAs was assayed in U87MG transfected for 24 h with the plasmid vector expressing circSMARCA5 or with an empty pcDNA3.1 vector (NC) through real-time PCR, by using two different sets of TaqMan^®^ Array MicroRNA Cards (A and B) (see Section 4). As shown in Figure 1A and Figure S1, a total of 11 and 17 miRNAs were differentially expressed (DE) between U87MG overexpressing circSMARCA5 and NCs, in Cards A and B, respectively. Among DE miRNAs, 15 were upregulated and 13 downregulated in U87MG overexpressing circSMARCA5 as compared to NCs. Analysis performed with DIANA miRPath showed an involvement of DE miRNAs in biological processes (BPs) and pathways related to glioma (Figure 1B,C; Table S1).

### 2.2. miRNAs 126-3p, 515-5p, and 1257 Are Upregulated upon circSMARCA5 Overexpression in U87MG

Based on our previous characterization of circSMARCA5 as TS in GBM, we focused on DE miRNAs that showed, upon circSMARC5 overexpression: (i) a fold-change (FC) of at least 1.5 and (ii) an upregulation or downregulation associated with a known TS or oncogenic function in GBM (or other neoplasia), respectively. These criteria allowed us to filter six DE miRNAs (five upregulated TS RNAs (miR-126-3p, miR-144-5p, miR-331-3p, miR-515-5p, and miR-1257) and one downregulated onco-miRNA (miR-517a-3p)) to be further analysed (Table 1).

**Figure 2:**
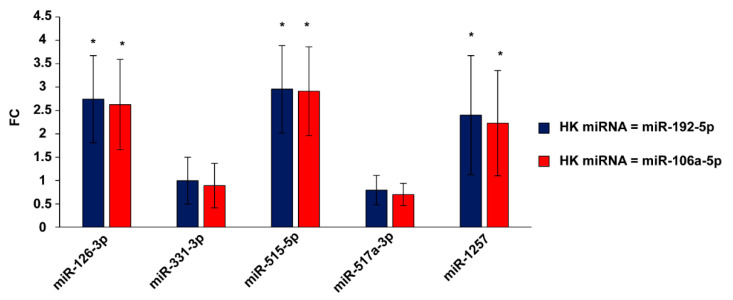
Expression of candidate DE miRNAs in U87MG overexpressing circSMARCA5. Data are reported as fold-change (FC) of the expression of candidate DE miRNAs in U87MG transfected with pcDNA3_circSMARCA5 vs. NCs. Red and blue bars show FC calculated by using miR-106a-5p and miR-192-5p as endogenous control (HK), respectively. The * *p*-value < 0.05 (*n* = 3, Student’s *t*-test).

Upregulation of miRNAs 126-3p, 515-5p, and 1257 in U87MG overexpressing circSMARCA5 was validated through single TaqMan^®^ assays; miR-144-5p was not detected in any sample following real-time PCR; expression of miR-331-3p and miR-517a-3p did not significantly change in U87MG overexpressing circSMARCA5 as compared to NCs (Figure 2).

### 2.3. miRNAs 126-3p and 515-5p Are Downregulated in GBM Biopsies and Their Expression Positively Correlates with That of circSMARCA5

**Figure 3:**
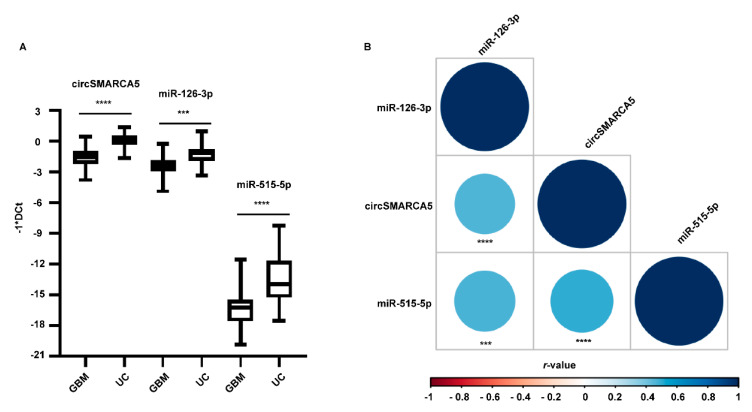
CircSMARCA5, miR-126-3p, and miR-515-5p are downregulated and positively correlated in GBM and UC biopsies. (**A**) Box and whisker plots representing the expression of circSMARCA5, miR-126-3p, and miR-515-5p in GBM and UC biopsies. Data are reported as −1*DCt. Statistical significance is indicated as *p*-value under the name of each transcript. (**B**) Correlogram showing correlations among circSMARCA5, miR-126-3p, and miR-515-5p. The colour of the circle is linked to the type of correlation (colours from light blue to dark blue and from light red to dark red are representative of positive and negative correlation, respectively); the size of the circle is inversely proportional to the *p*-value (the bigger is the circle, the less is the *p*-value). The *r*-values have been calculated by applying Spearman’s correlation test (*** *p*-value < 0.001, **** *p*-value < 0.0001).

To further analyse the correlation between circSMARCA5 and miRNAs 126-3p, 515-5p, and 1257, their expression was initially assayed in a training set cohort made up of five GBM and five UC samples. As shown in Figure S2, miRNAs 126-3p and 515-5p were significantly downregulated, while miR-1257 was upregulated in GBM vs. unaffected brain parenchyma (UC). The expression of miR-126-3p, miR-515-5p, and circSMARCA5 was then assayed in a validation set cohort made up of 38 GBM and 21 UC samples: circSMARCA5, miR-126-3p, and miR-515-5p were confirmed as downregulated in GBM biopsies, as compared to UCs (FC = −2.32, −1.59, and −2.82, *p*-values = 9.34 × 10^−9^, 7.9 × 10^−4^ and 2.97 × 10^−6^, Student’s *t*-test, respectively) (Figure 3A). MiR-126-3p and miR-515-5p were also positively correlated with circSMARCA5 (*r*-values = 0.49 and 0.50, *p*-values = 9 × 10^−5^ and 7 × 10^−5^, Spearman’s correlation test, respectively) (Figure 3B) and downregulated in all the GBM cell lines used in this study, as compared to brain cells from healthy donors (Figure S3).

### 2.4. IGFBP2 (Target of miR-126-3p), NRAS and ROCK1 (Targets of miR-515-5p) mRNAs Are Downregulated upon circSMARCA5 Overexpression in U87MG

**Figure 4:**
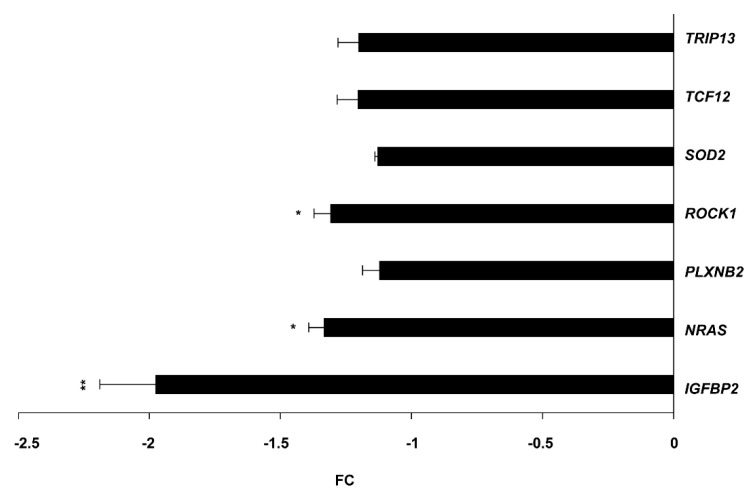
Expression of candidate DE miRNA targets in U87MG overexpressing circSMARCA5. Data are reported as fold-change (FC) of the expression of candidate miRNA targets in U87MG transfected with pcDNA3_circSMARCA5 *vs*. NCs. * *p*-value < 0.05, ** *p*-value < 0.01 (*n* = 3, Student’s *t*-test).

To widen our knowledge of the identified circSMARCA5/miR-126-3p/miR-515-5p axis, we searched for the targets of the two candidate miRNAs. A manual literature search allowed us to initially select 17 validated targets (9 of miR-126-3p and 8 of miR-515-5p) in GBM or other neoplasias (Table S2). This first selection was followed by the analysis of the Tumor Cancer Gene Atlas (TCGA), whose data were retrieved from the University of Alabama Cancer Database (UALCAN) to search for upregulated targets in GBM as compared to UCs. Based on TCGA data, we focused on eight mRNA targets (*IGFBP2*, *NRAS*, *PLXNB2*, *ROCK1*, *SOD2*, *TCF12*, *TRIP13*, and *VCAM1*) (Table 2), whose expression was first assayed in U87MG overexpressing circSMARCA5. Among the assayed targets, *IGFBP2*, *NRAS,* and *ROCK1* mRNAs were significantly downregulated (FC *IGFBP2*, *NRAS, ROCK1* = −1.97, −1.33, and −1.31, respectively) (Figure 4).

### 2.5. IGFBP2 and NRAS mRNAs Are Upregulated in GBM Biopsies and Cell Lines with Respect to UCs and Their Expression Negatively Correlates with That of circSMARCA5

**Figure 5:**
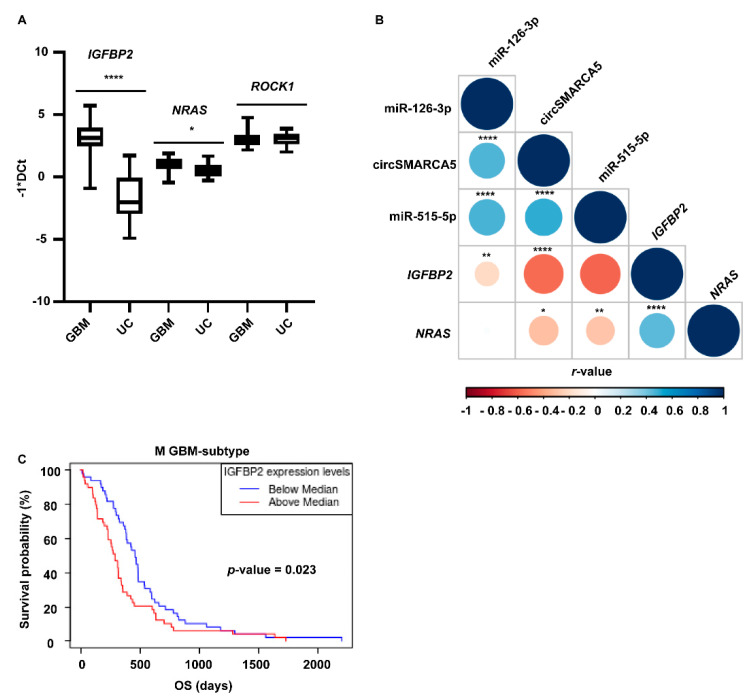
Expression of DE miRNA targets in GBM and UC biopsies. (**A**) Box and whisker plots representing the expression of *IGFBP2*, *NRAS,* and *ROCK1* mRNAs in GBM and UC biopsies. Data are reported as −1*DCt. Statistical significance is indicated as *p*-value under the name of each transcript. (**B**) Correlogram showing correlations among circSMARCA5, miR-126-3p, miR-515-5p, *IGFBP2*, and *NRAS*. The colour of the circle is linked to the type of correlation (colours from light blue to dark blue and from light red to dark red are representative of positive and negative correlation, respectively); the size of the circle is inversely proportional to the *p*-value (the bigger is the circle, the less is the *p*-value). *r*-values have been calculated by applying Spearman’s correlation test (**** *p*-value < 0.0001, ** *p*-value < 0.01, * *p*-value < 0.05). (**C**) Kaplan–Meier overall survival (OS) curves of Mesenchymal (M) GBM-subtype patients, based on the expression of IGFBP2. Data were retrieved from Glioblastoma Bio Discovery Portal (GBM-BioDP).

The expression of *IGFBP2*, *NRAS,* and *ROCK1* mRNAs was then assayed in the same validation set cohort (made of 38 GBM and 21 UC samples) used to verify the differential expression of candidate DE miRNAs. *IGFBP2* and *NRAS* were upregulated in GBM biopsies and cell lines with respect to UCs (Figure 5A and Figure S4) and their expression negatively correlated with that of circSMARCA5 (*r*-values = −0.58 and −0.30, *p*-values = 0 and 0.019, Spearman’s correlation test, respectively), and with that of their negative regulators, miR-126-3p and miR-515-5p, respectively (*r*-values = −0.36 and −0.34, *p*-values = 0.0066 and 0.010, Spearman’s correlation test, respectively) (Figure 5B). *IGFBP2* mRNA and protein were significantly upregulated in classical (C) and mesenchymal (M) GBM subtypes, when compared to the proneural (P) and neural (N) ones, based on TCGA data (Figures S5 and S6); *NRAS* mRNA was, instead, significantly upregulated in the N GBM subtype when compared to the other subtypes (Figure S7). These data are in agreement with the worst prognosis of M-subtype patients showing a higher *IGFBP2* mRNA expression (*p*-value = 0.023, Kaplan–Meier survival curve comparison) (Figure 5C). *IGFBP2* and *NRAS* mRNAs were positively correlated based on our dataset (*r*-value = 0.46, *p*-value = 0.00027) (Figure 5B), and GBM TCGA and normal brain cortex GTEx gene expression data (*r*-value = 0.76, *p*-value = 4.5 × 10^−52^) (Figure S8). To extend the pathway downstream to IGFBP2, we assayed the expression of the vascular endothelial growth factor A (*VEGFA*) mRNA (whose transcription is known to be activated by IGFBP2 protein that acts as an enhancer on its promoter—see Section 3) in (i) U87MG overexpressing circSMARCA5 and (ii) in the same validation set cohort used to verify the differential expression of *IGBP2* mRNA. *VEGFA* mRNA was significantly downregulated in U87MG upon circSMARCA5 overexpression and its expression positively correlated with that of *IGFBP2* in the validation set cohort used in this study (Figure S9).

### 2.6. Serine- and Arginine-Rich Splicing Factor 3 (SRSF3) Is Predicted to Bind Pre-miRNAs 126 and 515-1

To search for RNA motifs common to pre-miRNAs 126 (precursor of miR-126-3p) and 515-1 (precursor of miR-515-5p), their sequences were given as input to the MEME tool. Two motifs, *CUUCAA* and *CUCCAA*, were identified within pre-miR-126 and pre-mir-515-1 sequences, respectively (*p*-values = 4.72 × 10^−4^ and 2.12 × 10^−4^, respectively). Both motifs have been validated by other researchers to be bound by SRSF3, a known post-transcriptional regulator of miRNA processing (Table 3), and occur in the apical loop and the stem regions of pre-miRNAs 126 and 515-1 hairpins, respectively, according to the *RNAStructure* tool’s prediction (Figure S10).

## 3. Discussion

**Figure 6:**
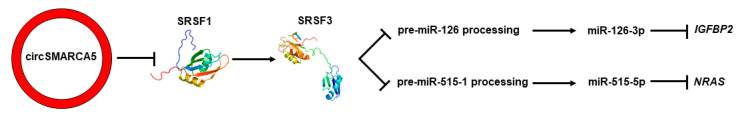
Schematic model of circSMARCA5-mediated upstream control of miRNAs 126-3p, 515-5p, and their mRNA targets *IGFBP2* and *NRAS*.

The pathway leading to the expression of miRNAs is very complex and tightly regulated by several factors at different steps [64]. Altered expression of the microRNAome in GBM has been extensively studied, although the molecular mechanisms steering specific miRNA dysregulation have been elucidated only in a few cases [65,66,67]. Numerous RBPs play a crucial role in the post-transcriptional processing of pri-miRNAs and pre-miRNAs [68]: among them, the splicing factor SRSF1 has been demonstrated to regulate the expression of several mature miRNAs by a cross-talk with the enzymes involved in the processing of their precursors, through mechanisms that have been only partially explained to date [69]. The interplay between circRNAs and miRNAs has been mainly described as a ceRNA network, in which circRNAs, including circSMARCA5, act as sponges for miRNAs [24,25,26,27,70,71,72,73,74]; however, circRNAs have not been yet reported as upstream regulators of miRNA expression, to the best of our knowledge. Because of their role as decoys for several RBPs, here we hypothesize that circRNAs may act as upstream epigenetic regulators of the miRNAome inside cells. In this work, we specifically investigated the circSMARCA5-mediated regulation of the miRNAome in GBM cells. We previously characterized circSMARCA5 as a TS circRNA in GBM and we demonstrated that it performs its function by sponging the RBP SRSF1 [22,23,75]. Our data ascertained that circSMARCA5 plays a role in the control of the miRNA expression inside GBM cells and that several dysregulated miRNAs upon circSMARCA5 overexpression are involved in glioma pathways. Further investigation led us to focus on miRNAs 126-3p and 515-5p: (i) both were upregulated in U87MG upon circSMARCA5 overexpression; (ii) their expression was positively correlated with that of circSMARCA5; (iii) they have been characterized as TS in GBM and additional cancers by other scholars [45,46,47,48,49,54,55,56,76,77,78,79,80,81,82,83,84,85,86,87]. CircSMARCA5-mediated upstream control of miRNAs 126-3p and 515-5p is also supported by: (i) the observed downregulation of their two selected mRNA targets, *IGFBP2* and *NRAS*, upon circSMARCA5 overexpression; (ii) positive correlation between the expression of *IGFBP2* and *NRAS* mRNAs; (iii) negative correlation between the expression of *IGFBP2* and *NRAS* mRNAs and circSMARCA5. CircSMARCA5 is also functionally linked to miRNAs 126-3p, 515-5p and their targets; indeed, similar to circSMARCA5 [21,22,23], miR-126-3p and its target *IGFBP2* are involved in GBM progression, by regulating cell migration, invasion [45,88,89,90], and angiogenesis [91,92]. To deepen the knowledge of the latter molecular axis, we also investigated the expression of *VEGFA* mRNA, both in U87MG overexpressing circSMARCA5 and in the validation cohort of GBM and UC biopsies. We previously showed that circSMARCA5 affects the ratio between pro- and anti-angiogenic isoforms of VEGFA mRNA in GBM by regulating alternative splicing of *VEGFA* pre-mRNA, tethering the splicing factor SRSF1 [22]. Here we showed that the amount of pan-*VEGFA* mRNA decreased in U87MG upon circSMARCA5 overexpression and that *VEGFA* and *IGFBP2* mRNAs were positively correlated. Unless here we focused on an axis involving (non-coding and coding) RNA molecules, data obtained on the expression of *VEGFA* mRNA suggest that IGFBP2 may be upstream regulated by circSMARCA5 also at the protein level: indeed, IGFBP2 was described as an enhancer for the transcription of VEGFA in neuroblastoma cells [93] and IGFBP2 and VEGFA were shown to be positively correlated at the protein level in GBM tissues [94]. MiR-126-3p can be also carried in biological fluids through EVs [95,96]: it would be interesting to investigate if and how the delivery of this molecule to cells at different sites from the bulk tumor play a role in the cancer progression and resistance to the current therapies. MiR-515-5p and its target NRAS are also known to be involved in GBM progression by regulating cell migration, growth [53,54,55,56,97], and angiogenesis [98,99]. In an attempt to find a link between the upstream regulator circSMARCA5 and the downstream-regulated intronic miRNAs 126-3p and 515-5p, we also searched for RBPs that may commonly bind and, potentially, regulate pre-miR-126 and pre-miR-515 processing. Our prediction allowed us to identify the splicing factor SRSF3 as an RBP that potentially binds both pre-miRNAs. We previously found that SRSF3 splicing is regulated by SRSF1 and, indirectly, by circSMARCA5 in GBM cells, where the pro-oncogenic full-length functional *SRSF3* mRNA isoform is overexpressed when compared to the truncated non-functional one [21]. SRSF3 has been described as a direct or indirect positive regulator of the processing of several pri-miRNAs such as pri-miRNAs 30a, 142, and miR-132/212, by interacting with a *CNNC* motif, recruiting DROSHA to the cleavage site, and enhancing the Microprocessor activity [100,101]. Based on our prediction, SRSF3 would interact with different motifs other than *CNNC* on pre-miRNA 126 and 515 sequences, paving the way to alternative mechanisms of SRSF3-mediated pri- and, eventually, pre-miRNA processing. Most specifically, based on our data, we speculate that in a GBM cell context and in particular for pre-miRNAs 126 and 515-1 processing, SRSF3 may function as a negative regulator (Figure 6). As previously reported for the RBP RNA binding fox-1 homolog 3 (RBFOX3), the same RBP can stimulate or block the processing of individual pri- or pre-miRNAs depending on the cell context and the specific miRNA precursor structure [102].

Collectively, our data suggest circSMARCA5 as an upstream regulator of the expression of TS miRNAs 126-3p and 515-5p and their downstream targets *IGFBP2* and *NRAS* mRNA in GBM cells, extending our knowledge on the disrupted tumor suppressive pathways mediated by circSMARCA5 in GBM cells. Prospectively, these pathways may be considered for targeted molecular therapeutic approaches, especially by using recently discovered genomic editing techniques [103].

## 4. Materials and Methods

### 4.1. Cell Lines and Biopsies

GBM cell lines A172, CAS-1, SNB-19, U251MG, and U87MG were cultured as described in the supplementary materials and methods. All cell lines were purchased from the Interlab Cell Line Collection (ICLC), located at the IRCCS Ospedale Policlinico San Martino, Genova, Italy. Cell lines were used between the 5th and 10th passage and their viability was assessed through the Trypan Blue Exclusion Test (ThermoFisher Scientific, Waltham, MA, USA) before each experiment, according to the protocol described by W Strober [104]. In total, 38 GBM and 21 UC biopsies were obtained, characterized by pathologists, and stored until their processing, as previously described [22]. Informed consent was signed by the patients before surgery. Demographic data of the patients enrolled in this study are summarized in Table S3. The entire study was performed according to the Declaration of Helsinki and approved by the local ethical Committee of the Azienda Ospedaliero-Universitaria “Policlinico-Vittorio Emanuele”, Catania, Italy (project identification code: 166/2015/PO, 17 December 2015).

### 4.2. Cell Transfection

U87MG cells were transfected by using lipofectamine 2000 (Thermofisher Scientific, Waltham, MA, USA), as previously described [21]. Briefly, 5 × 10^4^ cells were seeded in a 24-well plate, cultured for 24 h, and transfected with 500 ng of NC or the vector expressing circSMARCA5 (pcDNA3.1_circSMARCA5) for 24 h, according to the manufacturer’s instructions. Three replicates for each experimental condition were carried out and analysed accordingly. Data on circSMARCA5 overexpression upon transfection of U87MG are reported in Figure S11.

### 4.3. RNA Extraction

Total RNA was isolated through Trizol™ (Thermofisher Scientific, Waltham, MA, USA) in accordance with the manufacturer’s instructions and quantified by spectrophotometry, as previously described [105]. FirstChoice^®^ Human Brain Reference RNA (Ambion, Austin, TX, USA) was used as an additional UC.

### 4.4. microRNA TaqMan^®^ Arrays

MicroRNA TaqMan^®^ Arrays (Thermofisher Scientific, Waltham, MA, USA) were performed as previously described [106]. Briefly, 300 ng of total RNA isolated from each of the three biological replicates of U87MG, transfected for 24 h with the vector pcDNA3.1_circSMARCA5 or with NC, were reverse-transcribed into specific cDNAs of 748 microRNAs using the TaqMan™ MicroRNA Reverse Transcription Kit (ThermoFisher Scientific, Waltham, MA, USA) and the Megaplex™ RT Primers Human Pool A v 2.1 and Pool B v 3.0 (ThermoFisher Scientific, Waltham, MA, USA). The products of Megaplex™ reactions were then pre-amplified using the following kits: Megaplex™ PreAmp Primers, Human Pool A v. 2.1, and Human Pool B v. 3.0 (ThermoFisher Scientific, Waltham, MA, USA), according to the manufacturer’s instructions. The Megaplex™ PreAmp product of each sample was then loaded into an independent TaqMan^®^ Array MicroRNA Card A (in the case of samples reverse-transcribed and pre-amplified with pool A primers) or B (with samples reverse-transcribed and pre-amplified with pool B primers) (ThermoFisher Scientific, Waltham, MA, USA). PCR was performed using a QuantStudio™ 5 Real-Time PCR System (ThermoFisher Scientific, Waltham, MA, USA) (Figure S12).

### 4.5. Array Data Analysis

EDS files generated by the run of microRNA TaqMan^®^ Arrays were imported into the dashboard of a ThermoFisher cloud (https://apps.thermofisher.com/apps/spa/#/dashboard, accessed on November 2021) and then analysed through relative quantification application. Cycle thresholds (Ct_s_) were then calculated by the software and exported in a CSV file. Data from Cards A and B were analysed independently. Briefly, miRNAs that showed Ct_s_ > 35 in all the experimental conditions were considered too late and filtered out from data analysis. Correlations between the mean or median Ct value of each card and the Ct value of each miRNA were calculated to select candidate housekeeping (HK) miRNAs. A selection of 15 and 3 candidate HK miRNAs, among those showing the strongest correlation with the mean and median Ct values, were given as input to RefFinder (http://blooge.cn/RefFinder/?type=reference, accessed on November 2021) to select the best reference miRNAs within Cards A and B, respectively (Table S4). Reference miRNAs (miR-192-5p and miR-106a-5p for Card A; miR-452-3p and miR-19a-5p for Card B) were used to obtain DCt_s_, (Ct of the transcript of interest—Ct of the reference transcript). DCt_s_ of 190 and 58 miRNAs were given as input to the MeV (Multiple Experiment Viewer) tool v. 4.7.1 to retrieve significant DE miRNAs within Cards A and B, respectively. A graphical representation of microRNA TaqMan^®^ Arrays’ data analysis is reported in Figure S12.

### 4.6. Real-Time PCR

DE miRNAs were validated through single TaqMan™ microRNA assays. Briefly, 30 ng of total RNA were reverse transcribed through the TaqMan™ microRNA Reverse Transcription Kit (ThermoFisher Scientific, Waltham, MA, USA) by using miRNA-specific primers and then amplified through the TaqMan™ Universal Master Mix II (ThermoFisher Scientific, Waltham, MA, USA) by using specific TaqMan™ assays, according to the manufacturer’s instructions. Messenger RNAs of candidate miRNA targets were amplified by using the Power SYBR™ Green RNA-to-Ct™ 1-Step Kit (ThermoFisher Scientific, Waltham, MA, USA). PCRs were run in a QuantStudio™ 5 Real-Time PCR System (ThermoFisher Scientific, Waltham, MA, USA). Real-time PCR data were represented as −1*DCt_s_, FC, or log FC within the text (see supplementary methods for further explanation). The list of TaqMan™ assays and primers used in this study is shown in Table S5.

### 4.7. In Silico Analyses

BPs and pathways regulated by DE miRNAs were retrieved through DIANA miRPath 3.0 [107]: validated targets stored in TarBase v. 7.0 were selected to calculate BP and pathway enrichment. The expression of miRNA targets from the GBM TCGA dataset was retrieved through the UALCAN, GBM-BioDP, and Gene Expression Profiling Interactive Analysis (GEPIA) databases [108,109,110]. Multiple Em for Motif Elicitation (MEME) suite v. 5.4.1 [111] was used to retrieve RNA motifs within pre-miRNA sequences, using default parameters. ATtRACT database v. 0.99β [112] identified RBPs validated to bind specific RNA motifs. The RNA Structure tool [113] was used to calculate and visualize the secondary structures common to pre-miRNA sequences.

### 4.8. Statistical Analyses

Pearson’s and Spearman’s correlation tests were used to calculate correlations between the mean or median Ct value of each card and the Ct value of each miRNA, in order to select candidate housekeeping (HK) miRNAs. Only miRNAs showing correlation coefficients (*r*-values) ≥ 0.8 and *p*-value < 0.05 were considered as candidate DE miRNAs to be given as input to RefFinder. DE miRNAs were calculated through the Significance Analysis of Microarray (SAM) method within MeV tool v. 4.7.1 [114]. Only miRNAs reporting *q*-values = 0 were considered DE. Correlation tests and statistical significance were performed and calculated through GraphPad Prism v. 8.0.2. Student’s *t*-test was used to identify DE miRNAs and targets after single real-time PCR assays; for this, *p*-values < 0.05 were considered significant.

## Figures and Tables

**Table 1:** Upregulated TS miRNAs and downregulated onco-miRNAs chosen as candidate DE miRNAs to be further analysed through single TaqMan^®^ assays.

DE miRNA	Expression upon circSMARCA5 OE	Role of miRNA in GBM or Other Tumors	Reference from Literature
miR-126-3p	Upregulated	TS-miRNA	[45,46,47,48,49]
miR-144-5p	Upregulated	TS-miRNA	[50]
miR-331-3p	Upregulated	TS-miRNA	[51,52]
miR-515-5p	Upregulated	TS-miRNA	[53,54,55,56]
miR-517a-3p	Downregulated	Onco-miRNA	[57,58]
miR-1257	Upregulated	TS-miRNA	[59,60]

**Table 2:** Selected targets of miRNAs 126-3p and 515-5p. Data reported in the table have been retrieved from UALCAN database.

DE miRNA	Target	FC GBM vs. UC (UALCAN-TCGA)	*p*-Value (UALCAN-TCGA)
miR-126-3p	*IGFBP2*	43.64593019	<10^−12^
miR-126-3p	*PLXNB2*	2.135723573	0
miR-126-3p	*VCAM-1*	7.528862275	0
miR-515-5p	*NRAS*	1.878510556	0.0011
miR-515-5p	*ROCK1*	1.726470588	0.0575
miR-515-5p	*SOD2*	2.854586107	1.65 × 10^−12^
miR-515-5p	*TCF12*	5.298487733	0.0204
miR-515-5p	*TRIP13*	4.796287482	3.05 × 10^−9^

**Table 3:** Experimentally validated interactions between RBPs and RNA motifs *CUUCAA* and *CUCCAA* identified within pre-miR-126 and pre-mir-515-1 sequences.

Gene Name	RNA Motif	Reference	Q-Score (*ATtRACT*)
SRSF2	GG***CUCCAA***	[61]	0.001327
SRSF3	UU***CUCCAA***	[62]	0.000743
SRSF3	UA***CUUCAA***	[62]	0.014075
SRSF3	***CUUCAA***C	[62,63]	1
SRSF3	UU***CUUCAA***	[62]	0.012513

## Data Availability

The datasets used and/or analysed during the current study are available from the corresponding author upon reasonable request.

## Supplementary Materials

The following supporting information can be downloaded at: https://www.mdpi.com/article/10.3390/ijms232213676/s1. References [47,53,54,55,56,115,116,117,118,119,120,121,122] are cited in the supplementary materials.
