# Supplementary material for: circSMARCA5 Is an Upstream Regulator of the Expression of miR-126-3p, miR-515-5p, and Their mRNA Targets, Insulin-like Growth Factor Binding Protein 2 (IGFBP2) and NRAS Proto-Oncogene, GTPase (NRAS) in Glioblastoma
Source: Int J Mol Sci. 2022 Nov 8;23(22):13676. doi: 10.3390/ijms232213676 (PMC9690846; doi:10.3390/ijms232213676)
Supplement: Supplementary file 1 [file ijms-23-13676-s001.zip › ijms-1976107-supplementary.pdf]

Supplementary file

Supplementary Materials and Methods

Cell cultures

GBM Cell line	Cell culture medium
A172	Dulbecco's Modified Eagle's Medium (DMEM) with 4.5 g/L glucose (Sigma-Aldrich, Saint-Louis, MO, USA); 1% Penicillin/Streptomycin (P/S) (100X) (Carlo Erba Reagents, Milan, Italy); 10% Fetal bovine serum (FBS) (Carlo Erba Reagents); 1% Sodium Pyruvate (Na^{+}Pyr) (100 mM) (ThermoFisher Scientific); 1% L-Glutamine (L-Glut) (200 mM) (ThermoFisher Scientific)
CAS-1	DMEM with 1 g/L glucose (Sigma-Aldrich); 1% P/S (100X) (Carlo Erba Reagents); 10% FBS; 1% L-Glut (200 mM) (ThermoFisher Scientific)
SNB-19	DMEM with 1 g/L glucose (Sigma-Aldrich); 1% P/S (100X) (Carlo Erba Reagents); 10% FBS; 1% L-Glut (200 mM) (ThermoFisher Scientific)
U87MG	DMEM with 1 g/L glucose (Sigma-Aldrich); 1% P/S (100X) (Carlo Erba Reagents); 10% FBS; 1% Na^{+}Pyr (100 mM) (ThermoFisher Scientific); 1% MEM Non-Essential Amino Acids Solution (NEAA) (100X) (ThermoFisher Scientific)
U251MG	Minimum Essential Medium Eagle (MEM) (Sigma-Aldrich); 10% FBS; 1% L-Glut (200 mM) (ThermoFisher Scientific); 1% NEAA (100X) (ThermoFisher Scientific); 1% Na+Pyr (100 mM) (ThermoFisher Scientific)

Cells were grown at 37°C, 5% CO_2.

Real-time PCR data representation

Data of the heatmap showing the expression of DE miRNAs retrieved from microRNA TaqMan® arrays (Figure 1A) and of the box and whisker plots (Figures 3, 5, S2) were reported as -1*DCts. This representation allows to depict the expression of DE miRNAs within each experimental replicate (in the case of the heatmap in Figure 1A) or the distribution of the expression of candidate DE miRNAs or their selected targets within GBM and UC tissues (in the case of box and whisker plots in Figures 3, 5, S2). DCt values were multiplied for "-1", instead to be reported as is, to render the interpretation of the data more intuitive: lower -1*DCt values equal to lower expression; higher -1*DCt values equal to higher expression. FC was calculated according to the $2^{\wedge\text{-DDCt}}$ method by Livak et al. [1]. FCs were graphed to report relative quantification of the gene expression of single transcripts in GBM cell lines with respect to controls (as reported in Figures 2, 4, S4, S9, S11). Relative quantification hase been graphed as logarithm of the FC (Log FC) instead of FC in case of a wider range of FC values for different transcripts shown in a same graph (as in the case of Figures S1, S3).

Supplementary Tables

BP	*p*-value	#genes	#miRNAs
mitotic cell cycle	0	116	5
RNA metabolic process	0	81	5
nucleobase-containing compound catabolic process	0	214	5
response to stress	0	448	6
epidermal growth factor receptor signaling pathway	0	73	6
catabolic process	0	434	6
gene expression	0	221	6
mRNA metabolic process	0	77	6
membrane organization	0	154	6
cellular protein modification process	0	531	7
viral process	0	185	7
Fc-epsilon receptor signaling pathway	0	64	7
small molecule metabolic process	0	473	7
symbiosis encompassing mutualism through parasitism	0	202	7
neurotrophin TRK receptor signaling pathway	0	89	7
biological_process	0	2673	8
biosynthetic process	0	864	8
cellular nitrogen compound metabolic process	0	1056	9
macromolecular complex assembly	1.11E-16	187	5
fibroblast growth factor receptor signaling pathway	1.11E-16	68	6
cellular protein metabolic process	1.11E-15	103	4
cell death	9.33E-15	199	5
cellular component assembly	3.99E-14	245	5
blood coagulation	1.34E-11	100	6
transcription DNA-templated	1.36E-09	363	3
protein complex assembly	3.81E-09	149	5
transcription initiation from RNA polymerase II promoter	1.31E-08	64	4
platelet activation	5.94E-08	49	5
DNA metabolic process	4.05E-07	131	3
phosphatidylinositol-mediated signaling	3.47E-06	38	4
viral life cycle	9.14E-06	37	5
immune system process	1.73E-05	193	4
activation of signaling protein activity involved in unfolded protein response	1.91E-05	24	4
Fc-gamma receptor signaling pathway involved in phagocytosis	2.36E-05	24	4
innate immune response	4.45E-05	102	3
insulin receptor signaling pathway	0.000257	38	3
cellular lipid metabolic process	0.000272	37	3
transforming growth factor beta receptor signaling pathway	0.000486	55	4
positive regulation of protein insertion into mitochondrial membrane involved in apoptotic signaling pathway	0.00066	14	3
nuclear-transcribed mRNA catabolic process, deadenylation-dependent decay	0.001442	19	2
intrinsic apoptotic signaling pathway	0.001528	22	3
protein N-linked glycosylation via asparagine	0.008387	24	3

intracellular transport of virus	0.008913	11	3
generation of precursor metabolites and energy	0.009188	45	2
nucleocytoplasmic transport	0.015385	74	3
regulation of transcription from RNA polymerase II promoter in response to hypoxia	0.025085	12	3
RNA splicing	0.025458	67	4
chromatin organization	0.029824	23	2
cell cycle	0.032523	154	3
post-translational protein modification	0.042609	25	2
transcription DNA-templated	0	492	3
nucleic acid binding transcription factor activity	0	247	5
epidermal growth factor receptor signaling pathway	0	75	5
RNA metabolic process	0	88	5
mRNA metabolic process	0	83	5
nucleobase-containing compound catabolic process	0	240	5
small molecule metabolic process	0	518	5
membrane organization	0	181	5
mitotic cell cycle	0	133	6
cell death	0	249	6
catabolic process	0	497	6
Fc-epsilon receptor signaling pathway	0	69	6
cellular protein metabolic process	0	153	6
symbiosis encompassing mutualism through parasitism	0	190	6
poly(A) RNA binding	0	437	6
RNA binding	0	540	7
cellular protein modification process	0	642	7
response to stress	0	570	7
viral process	0	173	7
neurotrophin TRK receptor signaling pathway	0	102	7
protein binding transcription factor activity	0	171	8
molecular_function	0	3450	8
cellular_component	0	3473	8
nucleoplasm	0	383	8
cytosol	0	767	8
biological_process	0	3361	8
biosynthetic process	0	1075	8
gene expression	0	247	8
enzyme binding	0	416	9
cellular nitrogen compound metabolic process	0	1340	9
ion binding	0	1426	9
protein complex	0	990	9
organelle	0	2571	10
cellular component assembly	1.11E-16	318	6
macromolecular complex assembly	2.22E-16	217	4
intrinsic apoptotic signaling pathway	4.27E-13	33	5
DNA metabolic process	6.75E-13	193	4
blood coagulation	2.30E-12	124	5
fibroblast growth factor receptor signaling pathway	2.91E-12	63	4

protein complex assembly	1.54E-09	159	3
cytoskeletal protein binding	1.26E-08	155	4
immune system process	1.57E-07	317	5
positive regulation of protein insertion into mitochondrial membrane involved in apoptotic signaling pathway	3.21E-07	15	3
mRNA processing	6.84E-07	118	4
phosphatidylinositol-mediated signaling	8.46E-07	41	5
chromatin organization	4.55E-06	40	2
innate immune response	5.67E-06	155	5
RNA splicing	6.28E-06	76	4
post-translational protein modification	1.87E-05	49	3
enzyme regulator activity	2.38E-05	175	6
protein N-linked glycosylation via asparagine	6.80E-05	36	3
Fc-gamma receptor signaling pathway involved in phagocytosis	8.21E-05	26	4
vesicle-mediated transport	0.000135	186	4
TRIF-dependent toll-like receptor signaling pathway	0.000306	23	3
insulin receptor signaling pathway	0.000424	41	2
G2/M transition of mitotic cell cycle	0.000723	41	3
transcription initiation from RNA polymerase II promoter	0.000862	46	2
regulation of ubiquitin-protein ligase activity involved in mitotic cell cycle	0.000902	20	2
cell junction organization	0.000974	37	3
platelet activation	0.00151	40	2
nuclear-transcribed mRNA catabolic process deadenylation-dependent decay	0.001638	26	3
DNA damage response signal transduction by p53 class mediator resulting in cell cycle arrest	0.001658	20	3
G1/S transition of mitotic cell cycle	0.001779	33	2
termination of RNA polymerase II transcription	0.001873	12	3
MyD88-independent toll-like receptor signaling pathway	0.002002	23	3
nucleocytoplasmic transport	0.00228	87	4
toll-like receptor 10 signaling pathway	0.002322	14	2
activation of signaling protein activity involved in unfolded protein response	0.003314	20	2
transforming growth factor beta receptor signaling pathway	0.003491	35	3
positive regulation of ubiquitin-protein ligase activity involved in mitotic cell cycle	0.004084	19	2
negative regulation of transcription from RNA polymerase II promoter	0.004807	137	2
microtubule organizing center	0.005126	84	2
cellular component disassembly involved in execution phase of apoptosis	0.005151	15	2
toll-like receptor TLR1:TLR2 signaling pathway	0.005269	14	2
toll-like receptor TLR6:TLR2 signaling pathway	0.005269	14	2
transcription from RNA polymerase II promoter	0.005672	68	3
activation of MAPKK activity	0.007435	24	2
mRNA splicing via spliceosome	0.009072	37	2
endosome	0.00946	115	2

toll-like receptor 5 signaling pathway	0.011013	14	2
toll-like receptor 3 signaling pathway	0.012502	23	3
transcription corepressor activity	0.012882	50	3
negative regulation of transcription DNA-templated	0.014306	164	3
anaphase-promoting complex-dependent proteasomal ubiquitin-dependent protein catabolic process	0.016159	21	2
transcription factor binding	0.01617	122	3
mRNA 3'-end processing	0.019622	11	2
positive regulation of transcription DNA-templated	0.020599	112	2
protein polyubiquitination	0.021583	41	3
stress-activated MAPK cascade	0.024626	17	3
toll-like receptor 4 signaling pathway	0.024729	24	3
protein targeting	0.027749	53	2
toll-like receptor 9 signaling pathway	0.03085	14	2
nuclear-transcribed mRNA catabolic process nonsense-mediated decay	0.038054	19	1
endoplasmic reticulum unfolded protein response	0.047635	29	2
cell cycle	0.049449	113	2

Table S1. Biological pathways (BP) significantly enriched, based on DIANA miRPath data analysis. In red and green are shown BPs regulated by candidate upregulated and downregulated miRNAs, respectively.

**DE miRNA**	**Candidate mRNA target**	**Type of cancer where the targeting has been described**	**Bibliographic reference**
miR-126-3p	*CRK*	Target of miR-126-3p and oncogene in lung cancer	[2]
miR-126-3p	*IGFBP2*	Target of miR-126-3p and oncogene in glioma	[3]
miR-126-3p	*IRS1*	Target of miR-126-3p and oncogene in glioma	[3]
miR-126-3p	*KRAS*	Target of miR-126-3p and oncogene in glioma	[3,4]
miR-126-3p	*LRP6*	Target of miR-126-3p and oncogene in hepatocellular carcinoma	[5]
miR-126-3p	*PLXNB2*	Target of miR-126-3p and oncogene in glioma	[6]
miR-126-3p	*SOX2*	Target of miR-126-3p and oncogene in glioma	[7]
miR-126-3p	*SPRED1*	Target of miR-126-3p and oncogene in hepatocellular carcinoma	[8]
miR-126-3p	*VCAM-1*	Target of miR-126-3p and oncogene in lung cancer	[2]
miR-515-5p	*MAPK4*	Target of miR-515-5p and oncogene in breast cancer	[9]
miR-515-5p	*MARK4*	Target of miR-515-5p and oncogene in glioma, breast and lung cancer	[10,11]
miR-515-5p	*NFE2L1*	Target of miR-515-5p and oncogene in glioma	[12]
miR-515-5p	*NRAS*	Target of miR-515-5p and oncogene in breast and lung cancer	[11]
miR-515-5p	*ROCK1*	Target of miR-515-5p and oncogene in glioma	[12]

miR-515-5p	*SOD2*	Target of miR-515-5p and oncogene in glioma	[13]
miR-515-5p	*TCF12*	Target of miR-515-5p and oncogene in glioma and ovarian cancer	[9]
miR-515-5p	*TRIP13*	Target of miR-515-5p and oncogene in prostate cancer	[9]

Table S2. List of validated candidate targets of miR-126-3p and miR-515-5p.

Sample	N	Mean Age (Years ± StdDev)	Sex	
			Male	Female
Fresh frozen GBM biopsies	38	65.4 ± 10.3	20	18
Fresh frozen UCs	21	64.9 ± 10.5	10	11

Table S3. Demographic data including sex and age of the study group.

Candidate HK miRNA	*r*-value (Pearson's correlation *vs* Mean Ct)	*r*-value (Pearson's correlation *vs* Median Ct)	*p*-value (Pearson's correlation *vs* Mean Ct)	*p*-value (Pearson's correlation *vs* Median Ct)
miR-192-5p	0.996	0.9858	****	****
miR-106a-5p	0.9929	0.9904	****	****
miR-374a-5p	0.9919	0.967	****	***
miR-155-5p	0.9904	0.947	****	**
miR-532-5p	0.9887	0.9828	****	****
miR-100-5p	0.981	0.9901	****	****
miR-17-5p	0.9809	0.9546	****	***
miR-28-5p	0.979	0.9545	***	***
let-7g-5p	0.977	0.9419	***	**
miR-660-5p	0.9767	0.9775	***	***
miR-361-5p	0.9763	0.9754	***	***
miR-25-3p	0.9757	0.9157	***	**
miR-222-3p	0.9756	0.984	***	****
miR-26a-5p	0.9754	0.9898	***	****
miR-15b-5p	0.9751	0.9714	***	***
miR-1203	0.9242	0.9029	**	*
miR-452-3p	0.9548	0.8758	**	*
miR-19a-5p	0.9301	0.8317	**	*

Table S4. Candidate endogenous control (HK) miRNAs given as input to RefFinder. In green and yellow are represented candidate HK miRNAs of Array Cards A and B, respectively. **** p-value < 0.0001, *** p-value < 0.001, ** p-value < 0.01, * p-value < 0.05.

**circRNA/miRNA/mRNA name**	**Forward primer sequence**	**Reverse primer sequence**	**TaqMan® Probe (Assay ID – ThermoFisher Scientific)**
circSMARCA5	ACAATGGATACAGAGTCAAGTGTT	CACATGTGTTGCTCCATGTCT	
miR-106a-5p			002169
miR-126-3p			002228
miR-144-5p			002148
miR-192-5p			000491
miR-331-3p			000545
miR-515-5p			001112
miR-517a-3p			002402
miR-1257			002910
U6			001973
IGFBP2	TGCACATCCCCAACTGTGAC	TGTAGAAGAGATGACACTCGGG	
NRAS	TGGGATTGCCATGTGTGGTG	AGGGTGTCAGTGCAGCTTG	
PLXNB2	CCTGGTGATCCCCATGAACC	ACCGGCTCCATGAACTTGAG	
ROCK1	AACATGCTGCTGGATAAATCTGG	TGTATCACATCGTACCATGCCT	
SOD2	TTTCAATAAGGAACGGGGACAC	GTGCTCCCACACATCAATCC	
TBP	ACTTGACCTAAAGACCATTGCA	GGCTCTCTTATCCTCATGATTACC	
TCF12	AGTTATCCATCTCCTAAGCCACC	AAGAATTGTGGGTCCCATCTTG	
TRIP13	AGCTGAATGAAGATGGCCCC	TACCAAGCTGTCCCAAAGCC	
VCAM1	CAGTAAGGCAGGCTGTAAAAGA	TGGAGCTGGTAGACCCTCG	

Table S5. Primer sequences and TaqMan® assay IDs used in this study. U6 was used as HK miRNAs to assay candidate miRNA expression in biopsies and GBM cell lines. *TBP* was used as HK to assay the expression of circSMARCA5 and the candidate miRNA targets.

Supplementary Figures

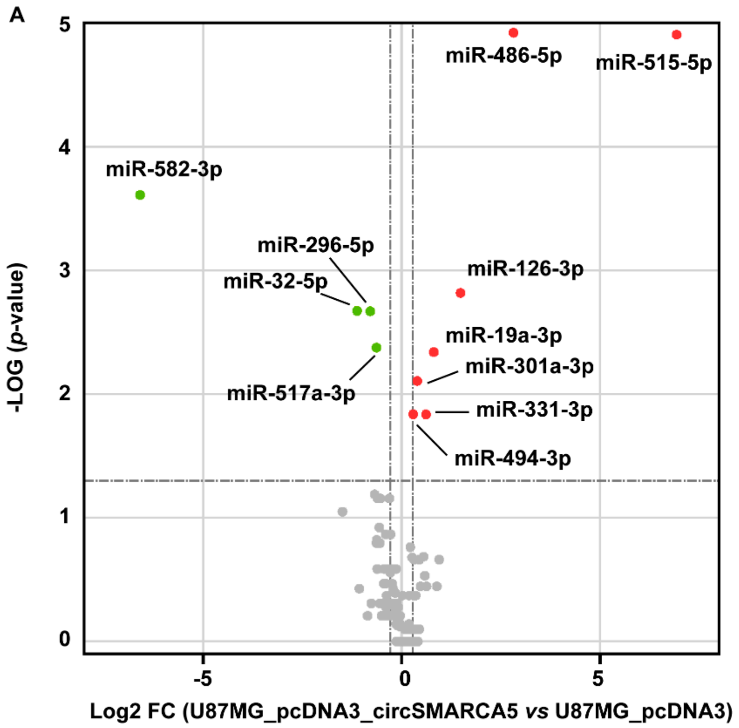

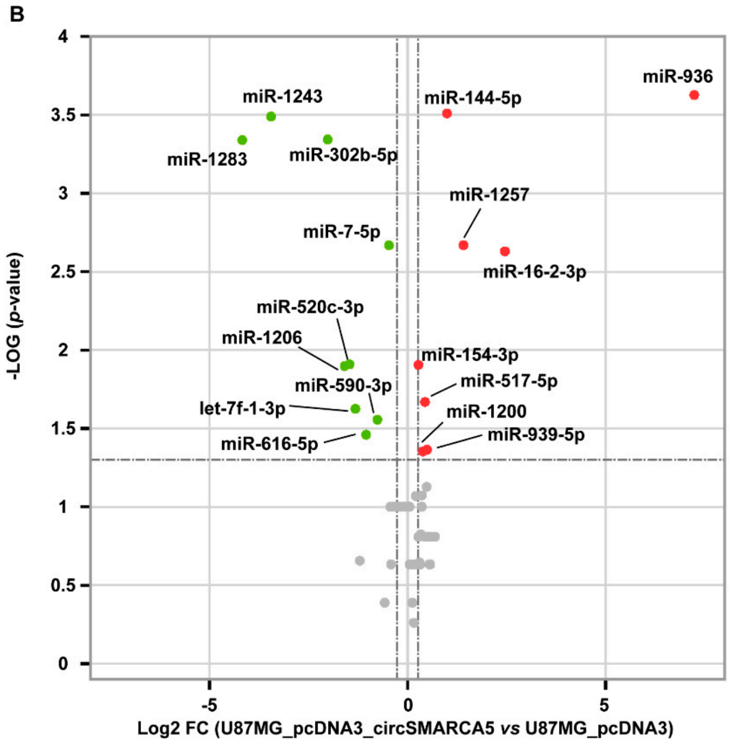

Figure S1. Volcano plots representing DE miRNAs retrieved from data analysis of TaqMan® Array MicroRNA Cards A (A) and B (B). Green and red dots represent statistically significant downregulated and upregulated DE miRNAs, respectively. Grey dots represent miRNAs that resulted not DE, based on SAM analysis. Volcano plots were generated by https://www.bioinformatics.com.cn/en, a free online platform for data analysis and visualization.

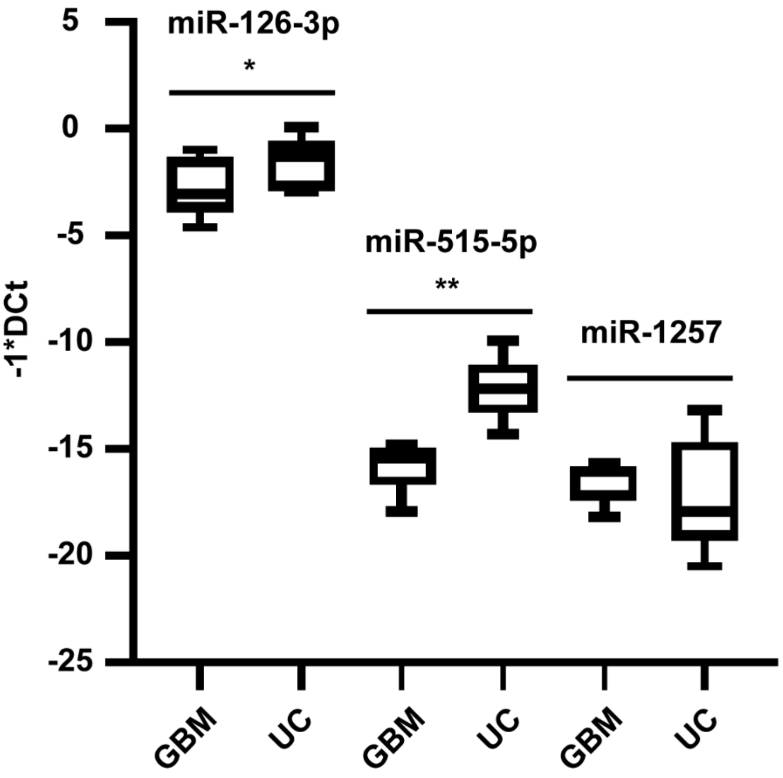

Figure S2. Box and whisker plots representing the expression of miR-126-3p, miR-515-5p and miR-1257 in GBM and UC biopsies. Data are reported as -1*DCt. * *p*-value < 0.05, ** *p*-value < 0.01 (n = 5, Stundent's *t*-test).

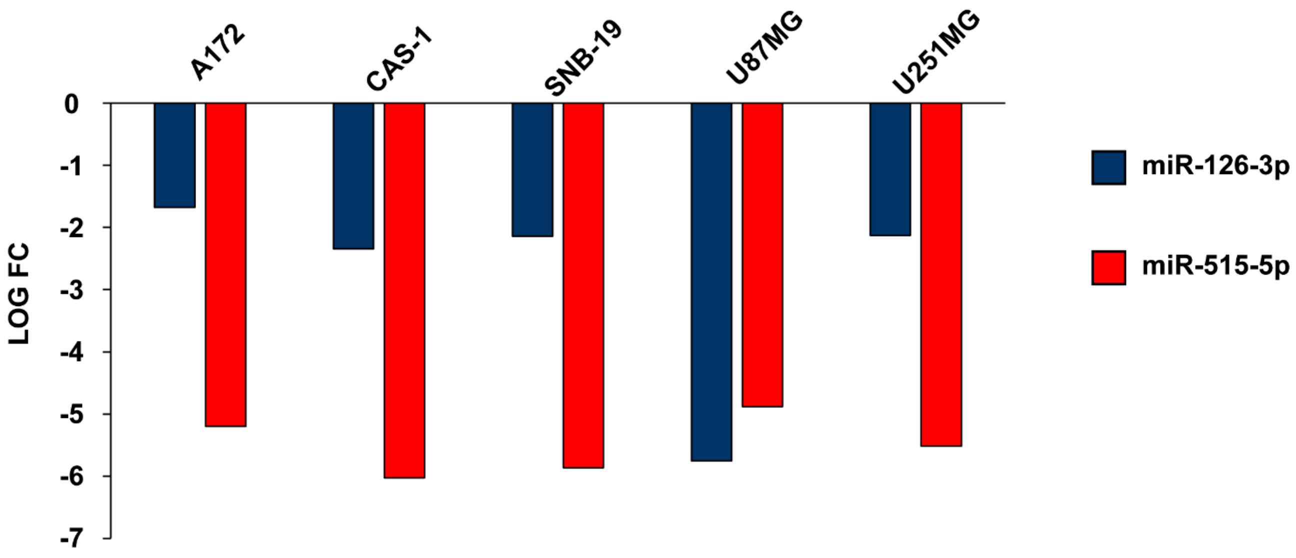

Figure S3. **Expression of candidate miR-126-3p (blue bars) and miR-515-5p (red bars) in GBM cell lines.** Data are reported as LOG fold-change (FC) of the expression of candidate miRNAs in each cell line *vs* FirstChoice® Human Brain Reference RNA (Ambion, Austin, TX, USA).

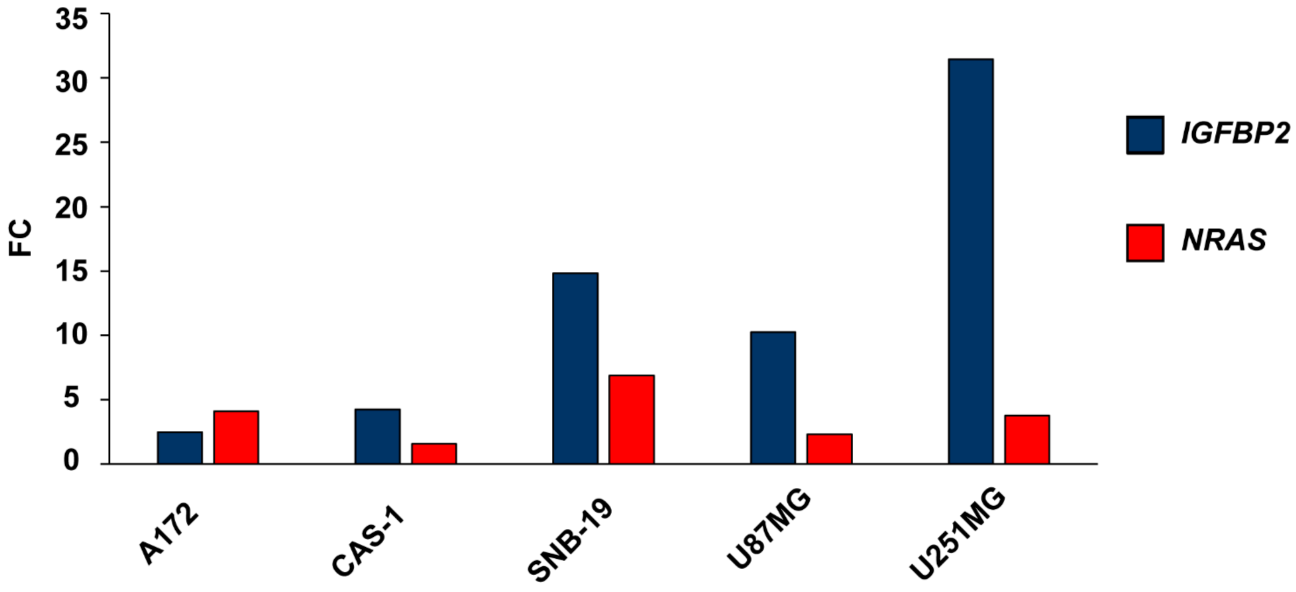

Figure S4. **Expression of *IGFBP2* (blue bars) and *NRAS* (red bars) mRNA in GBM cell lines.** Data are reported as fold-change (FC) of the expression of mRNA targets in each cell line *vs* FirstChoice® Human Brain Reference RNA (Ambion, Austin, TX, USA).

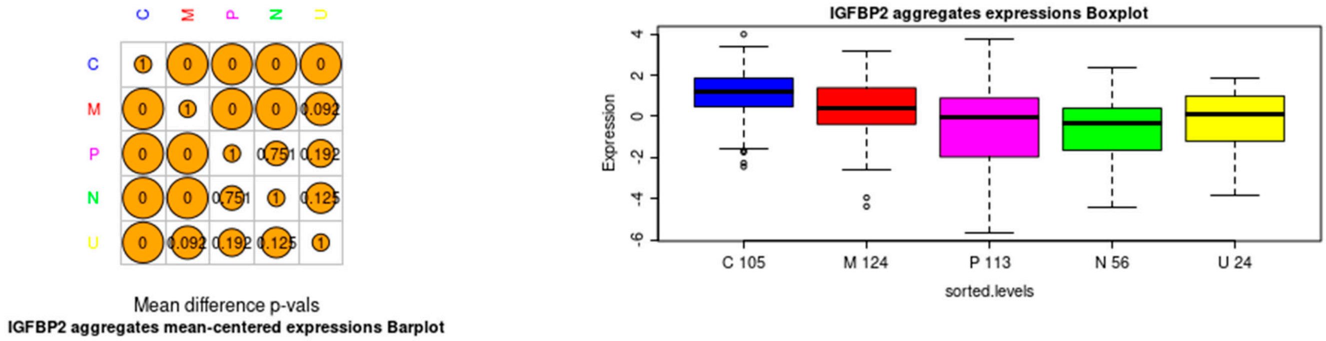

Figure S5. Expression of *IGFBP2* mRNA in Classical (C), Mesenchymal (M), Proneural (P), Neural (N) and Unclassified (U) GBM subtypes (data from GBM-BioDP).

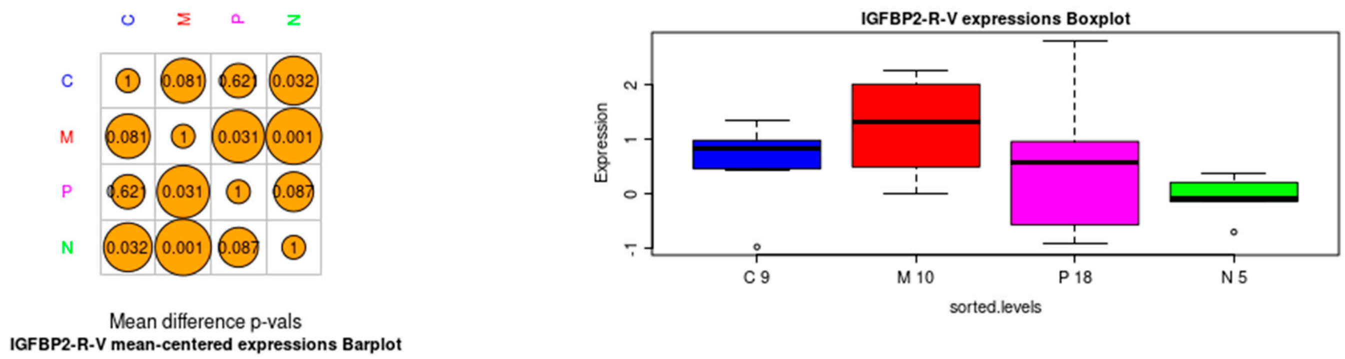

Figure S6. Expression of IGFBP2 protein in Classical (C), Mesenchymal (M), Proneural (P), Neural (N) GBM subtypes (data from GBM-BioDP).

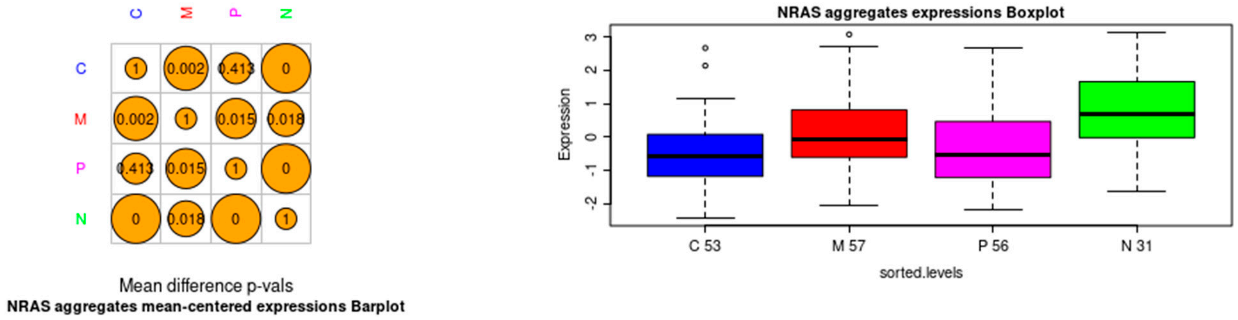

Figure S7. Expression of *NRAS* mRNA in Classical (C), Mesenchymal (M), Proneural (P), Neural (N) GBM subtypes (data from GBM-BioDP).

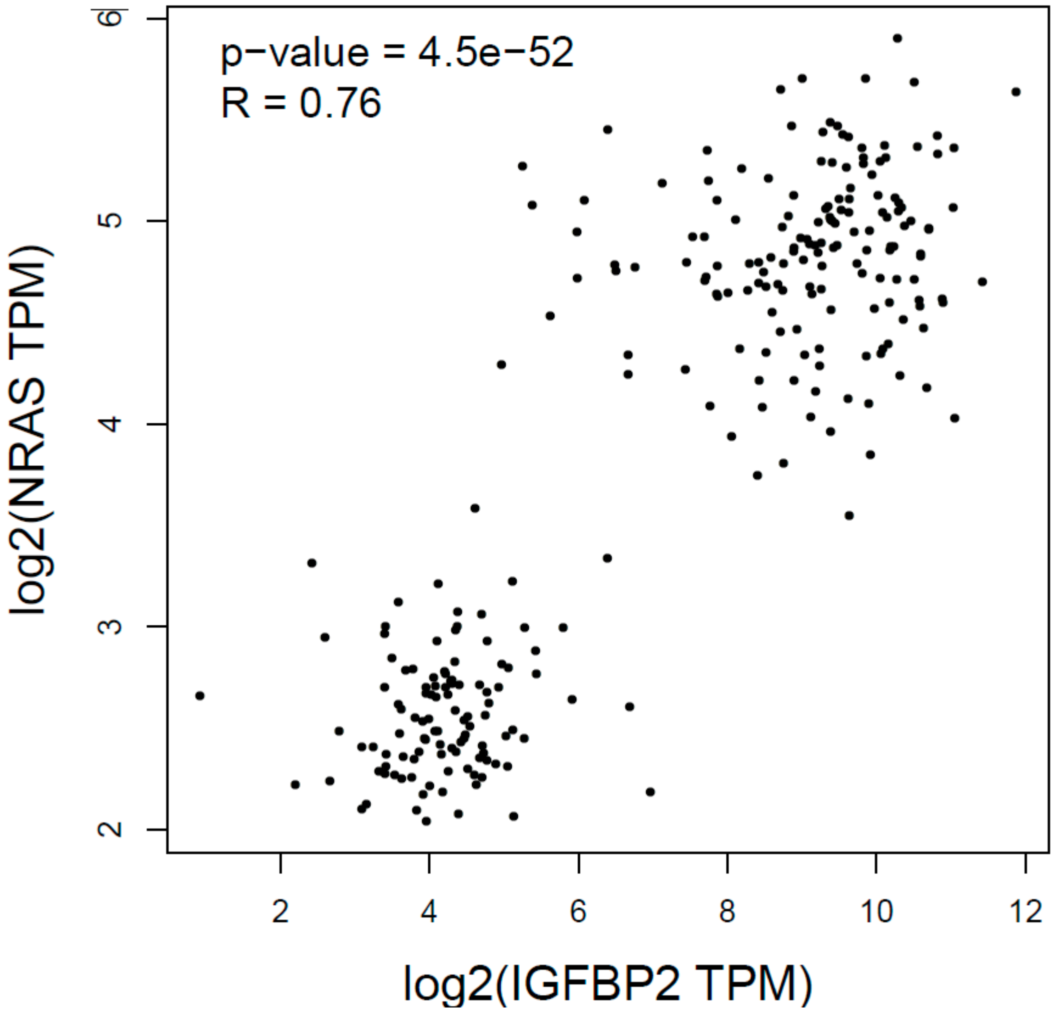

Figure S8. Scatter plot of the correlation between *IGFBP2* and *NRAS* mRNAs (data from GEPIA).

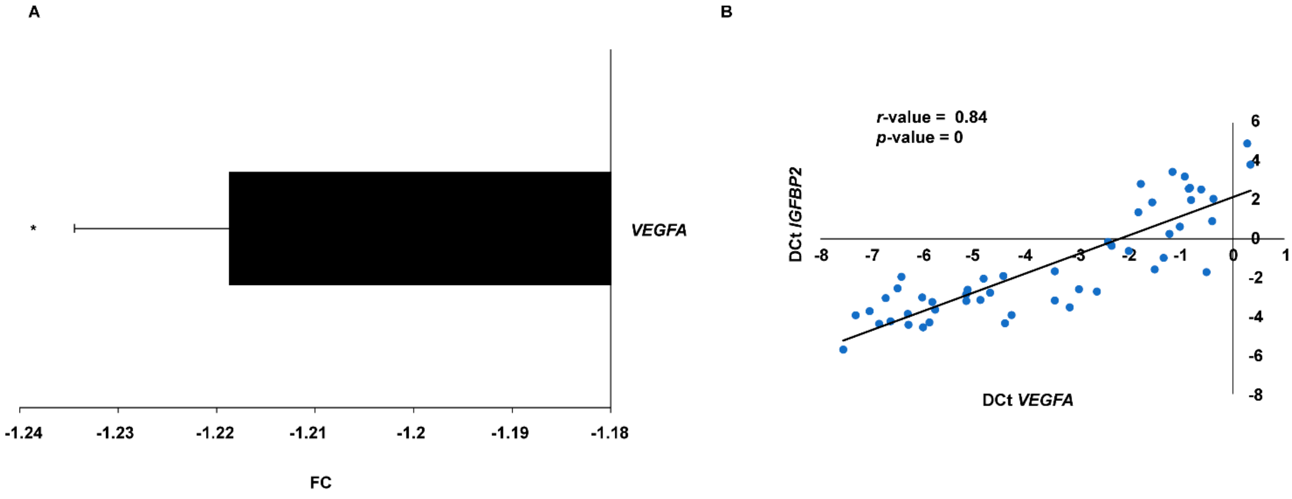

Figure S9. (A) *VEGFA* mRNA expression in U87MG overexpressing circSMARCA5. Data are reported as FC *vs* U87MG_pcDNA3 (* p-value < 0.05, n = 3, Student's t-test). (B) Scatter plot of the correlation between the expression of *VEGFA* and *IGFBP2* mRNA, calculated within the validation set cohort.

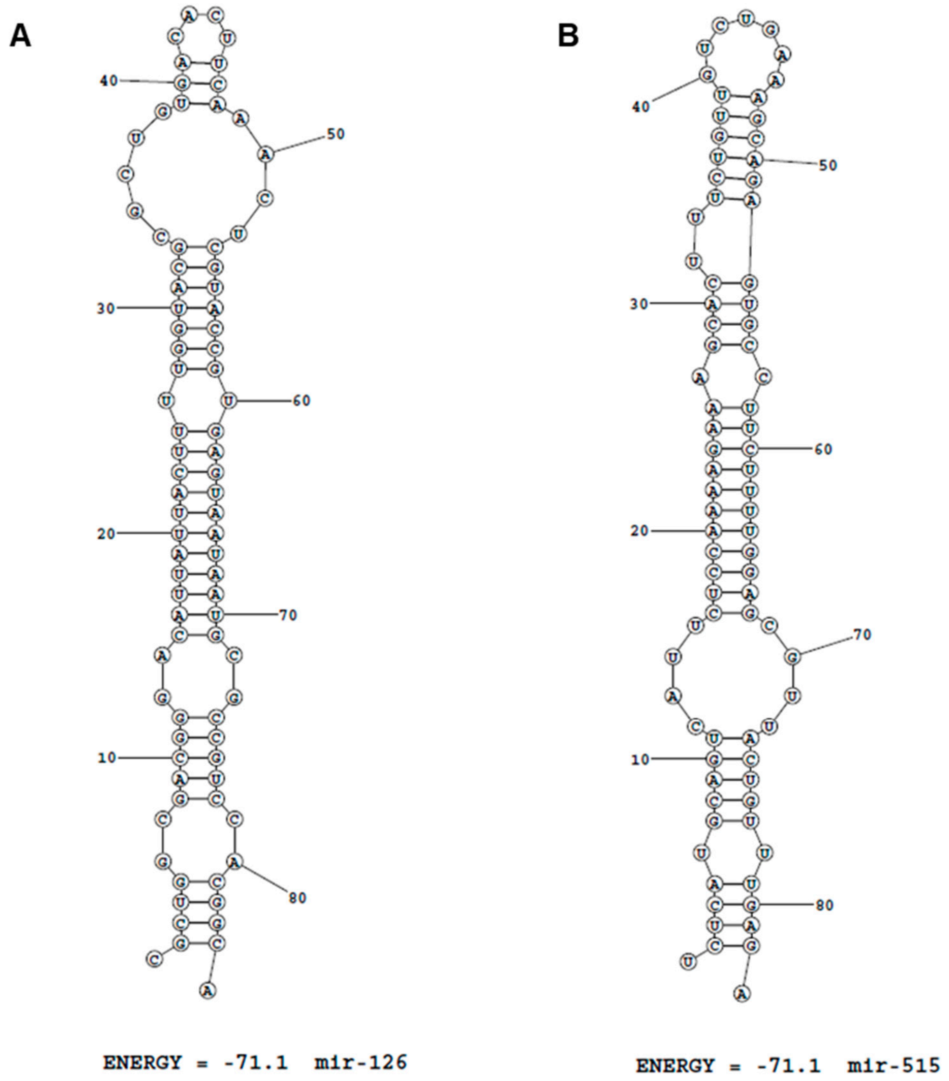

Figure S10. The most stable predicted secondary structures of pre-miRNAs 126 and 515-1, based on the tool *RNA Structure*.

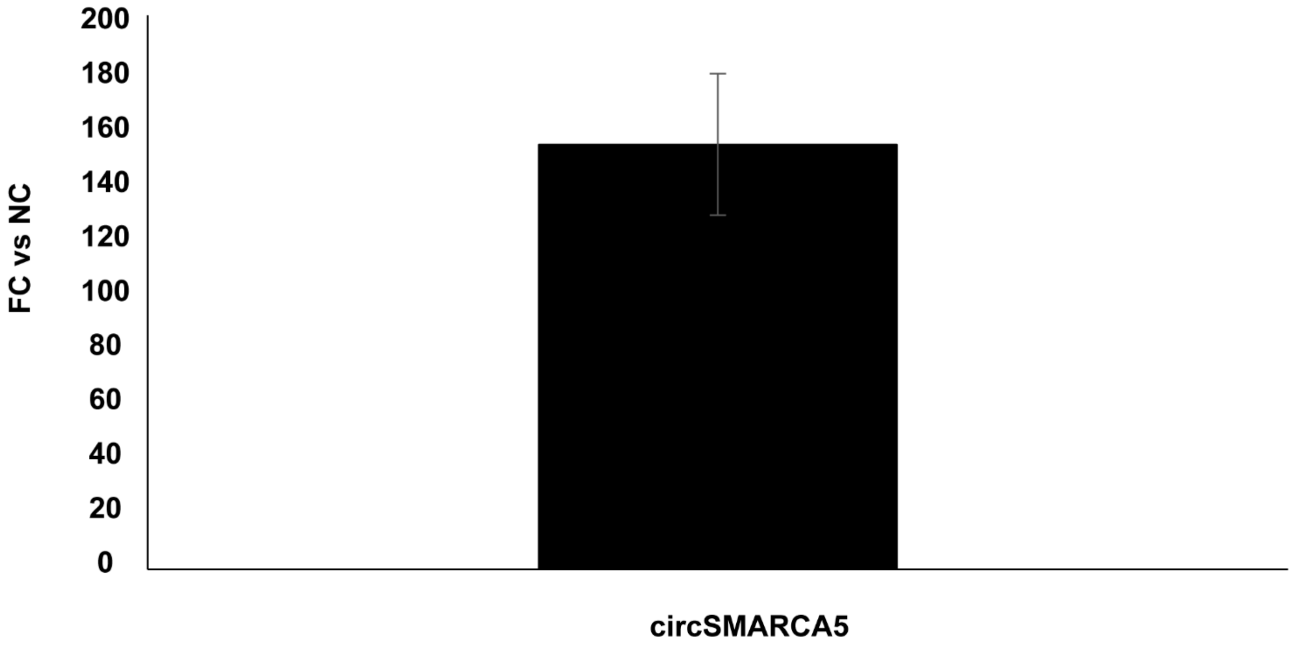

Figure S11. Expression of circSMARCA5 in U87MG transfected with pcDNA3_circSMARCA5. Data are reported as fold-change (FC) of the expression of circSMARCA5 in U87MG pcDNA3_circSMARCA5 vs U87MG pcDNA3.

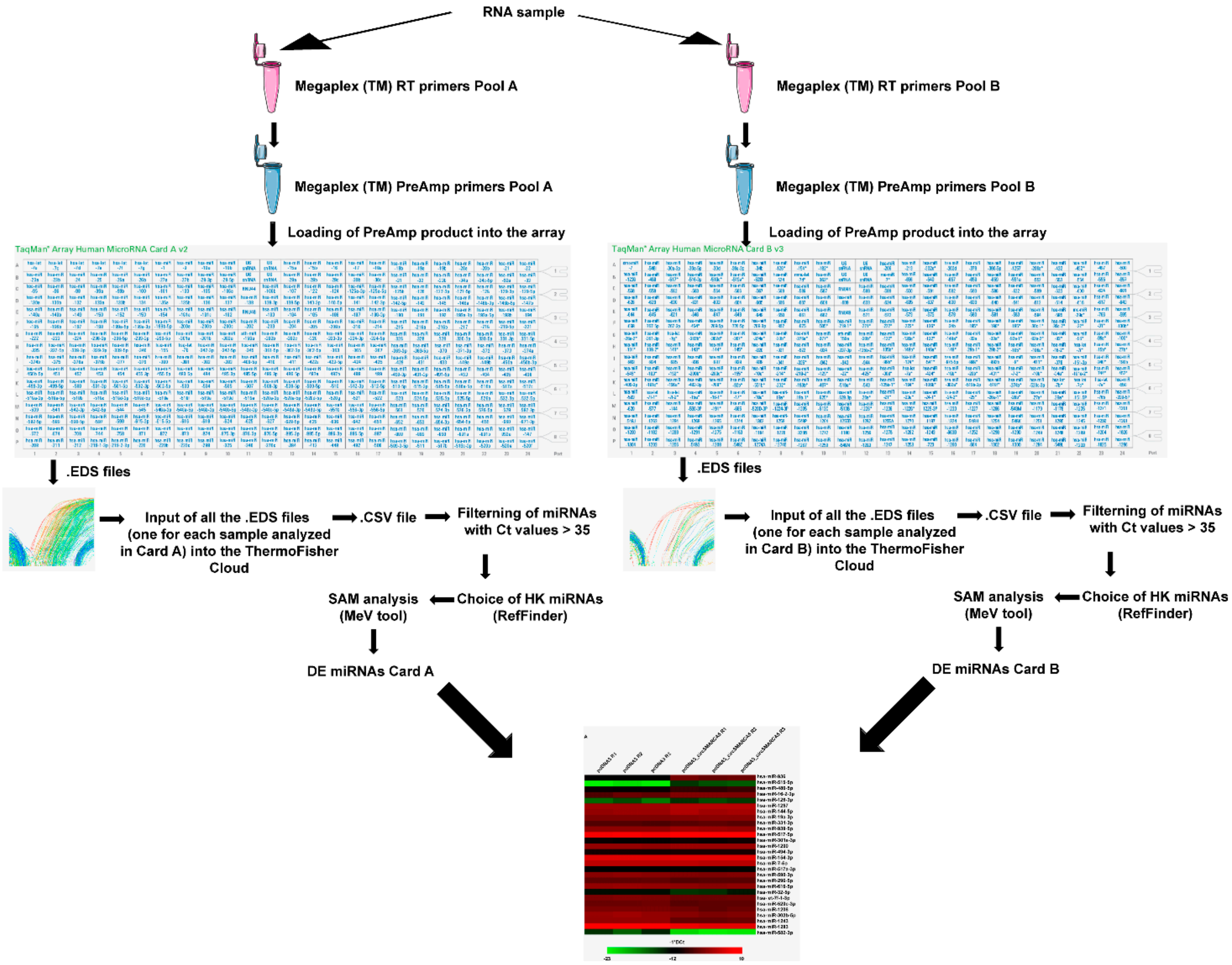

Figure S12. Graphical representation of microRNA TaqMan® Arrays' data analysis. The figure was partly generated using Servier Medical Art, provided by Servier, licensed under a Creative Commons Attribution 3.0 unported license.

Supplementary references

1. Livak, K.J.; Schmittgen, T.D. Analysis of relative gene expression data using real-time quantitative PCR and the 2(-Delta Delta C(T)) Method. *Methods* **2001**, *25*, 402-408, doi:10.1006/meth.2001.1262.
2. Han, I.B.; Kim, M.; Lee, S.H.; Kim, J.K.; Kim, S.H.; Chang, J.H.; Teng, Y.D. Down-regulation of MicroRNA-126 in Glioblastoma and its Correlation with Patient Prognosis: A Pilot Study. *Anticancer Res* **2016**, *36*, 6691-6697, doi:10.21873/anticanres.11280.
3. Rouigari, M.; Dehbashi, M.; Ghaedi, K.; Pourhossein, M. Targetome Analysis Revealed Involvement of MiR-126 in Neurotrophin Signaling Pathway: A Possible Role in Prevention of Glioma Development. *Cell J* **2018**, *20*, 150-156, doi:10.22074/cellj.2018.4901.
4. Chen, S.R.; Cai, W.P.; Dai, X.J.; Guo, A.S.; Chen, H.P.; Lin, G.S.; Lin, R.S. Research on miR-126 in glioma targeted regulation of PTEN/PI3K/Akt and MDM2-p53 pathways. *Eur Rev Med Pharmacol Sci* **2019**, *23*, 3461-3470, doi:10.26355/eurrev_201904_17711.
5. Du, C.; Lv, Z.; Cao, L.; Ding, C.; Gyabaah, O.A.; Xie, H.; Zhou, L.; Wu, J.; Zheng, S. MiR-126-3p suppresses tumor metastasis and angiogenesis of hepatocellular carcinoma by targeting LRP6 and PIK3R2. *J Transl Med* **2014**, *12*, 259, doi:10.1186/s12967-014-0259-1.
6. Xiang, G.; Cheng, Y. MiR-126-3p inhibits ovarian cancer proliferation and invasion via targeting PLXNB2. *Reprod Biol* **2018**, *18*, 218-224, doi:10.1016/j.repbio.2018.07.005.
7. Luo, W.; Yan, D.; Song, Z.; Zhu, X.; Liu, X.; Li, X.; Zhao, S. miR-126-3p sensitizes glioblastoma cells to temozolomide by inactivating Wnt/beta-catenin signaling via targeting SOX2. *Life Sci* **2019**, *226*, 98-106, doi:10.1016/j.lfs.2019.04.023.
8. Tan, W.; Lin, Z.; Chen, X.; Li, W.; Zhu, S.; Wei, Y.; Huo, L.; Chen, Y.; Shang, C. miR-126-3p contributes to sorafenib resistance in hepatocellular carcinoma via downregulating SPRED1. *Ann Transl Med* **2021**, *9*, 38, doi:10.21037/atm-20-2081.
9. Zheng, K.; Xie, H.; Wu, W.; Wen, X.; Zeng, Z.; Shi, Y. CircRNA PIP5K1A promotes the progression of glioma through upregulation of the TCF12/PI3K/AKT pathway by sponging miR-515-5p. *Cancer Cell Int* **2021**, *21*, 27, doi:10.1186/s12935-020-01699-6.
10. Naqvi, A.A.T.; Jairajpuri, D.S.; Hussain, A.; Hasan, G.M.; Alajmi, M.F.; Hassan, M.I. Impact of glioblastoma multiforme associated mutations on the structure and function of MAP/microtubule affinity regulating kinase 4. *J Biomol Struct Dyn* **2021**, *39*, 1781-1794, doi:10.1080/07391102.2020.1738959.
11. Pardo, O.E.; Castellano, L.; Munro, C.E.; Hu, Y.; Mauri, F.; Krell, J.; Lara, R.; Pinho, F.G.; Choudhury, T.; Frampton, A.E., et al. miR-515-5p controls cancer cell migration through MARK4 regulation. *EMBO Rep* **2016**, *17*, 570-584, doi:10.15252/embr.201540970.
12. Wang, Z.; Han, Y.; Li, Q.; Wang, B.; Ma, J. LncRNA DLGAP1-AS1 accelerates glioblastoma cell proliferation through targeting miR-515-5p/ROCK1/NFE2L1 axis and activating Wnt signaling pathway. *Brain Behav* **2021**, *11*, e2321, doi:10.1002/brb3.2321.
13. Zhang, Y.; Zhang, Y.; Wang, S.; Li, Q.; Cao, B.; Huang, B.; Wang, T.; Guo, R.; Liu, N. SP1-induced lncRNA ZFPM2 antisense RNA 1 (ZFPM2-AS1) aggravates glioma progression via the miR-515-5p/Superoxide dismutase 2 (SOD2) axis. *Bioengineered* **2021**, *12*, 2299-2310, doi:10.1080/21655979.2021.1934241.
